# Euthanasia in Mental Health. About a case

**DOI:** 10.1192/j.eurpsy.2022.924

**Published:** 2022-09-01

**Authors:** I. González Bocelo, I. Millan Lozano

**Affiliations:** 1 University Hospital La Paz, Psychiatry, MADRID, Spain; 2 Universitary Hopsital La paz, Psychiatrist, MADRID, Spain

**Keywords:** mental disorder, Euthanasia

## Abstract

**Introduction:**

Reflection on the impact of this law on people with a Mental health disorder is required. It is a complex issue, in which it must be considered that these disorders are very prevalent, can greatly affect the quality of life and are often resistant to treatment.

**Objectives:**

Explore the first request of this type and review aspects to consider in patients with mental health disorders.

**Methods:**

Collection of data from the first patient who requests it and review related aspects.

**Results:**

This is a patient with a personality disorder who presents significant suffering, in a chronic context, but at the same time makes the request in a moment of crisis as an impulsive gesture.

**Conclusions:**

Pending evolution, we consider that it is difficult to assess compliance with the requirements of the law in patients of this type and we recommend caution.

**Disclosure:**

No significant relationships.

